# The Case against Provisional Arrangements

**Published:** 1913-01-18

**Authors:** 


### SCHOOL CLINICS FOR LONDON.

### The Case Against Provisional Arrangements.

At a recent meeting of the London County Council the question of the establishment of school clinics was discussed in connection with a definite motion that the Council should proceed with the establishment of such centres. This motion was lost on a division by a small majority, but the discussion showed that there is a general feeling that the present arrangements for treatment of school children found to be defective at the routine examinations are not ideal. Nor, indeed, can anyone who has had experience of the manner in which such treatment is now carried out claim that the present arrangements can be allowed to continue indefinitely.

There is no co-ordination and comparatively no control at present. The provisional arrangements with the hospitals are open to grave objections not only on the score of efficiency, but also on economic grounds. It is stated that there is no reason to believe that the establishment of special school clinics, properly equipped and staffed by whole-time officers who are directly under the control of the Council's Public Health Department, will be more economical than the present arrangement, whereby the Council subsidises certain institutions and privately managed clinics. We do not agree that this is a fair statement. Nearly every clinic now existing in London is worked extravagantly, wastefully. Under proper supervision and control much might be done to secure uniformity of treatment and equality of expenditure. From the point of view of the parents, the establishment of school clinics would be far preferable to the existing system, which entails all the disadvantages of out-patient attendance and lax out-patient treatment.

A further argument is that our hospitals are not for the treatment of school children. They exist primarily for the relief of the poor, and, however much hospital secretaries and committee-men may countenance the continuance in their out-patient departments of work that should properly be relegated to school clinics, it seems to us that it is a short-sighted policy to permit such work to be done while there is a hope of securing, by refusing to allow such work, the establishment of duly equipped school clinics. Nor is the system of subsidising what may be called private practitioners' clinics a good one. Apart altogether from the question whether the treatment in such centres is adequate—and it is no secret that many school doctors consider that the treatment is not good—the interference with school work and the wasteful expenditure of time are the same in these clinics as in the hospital out-patient departments.

The real way out of the difficulty lies in the establishment of special school clinics. The London County Council has shown that it is fully alive to the urgent necessity of making the medical inspection of its schools as thorough as it can possibly be made. Its new policy of appointing a whole-time staff has had the best results, and will be even more successful in the future when the machine is running smoothly.

Medical inspection in the county is now second to none in the kingdom, but medical treatment still leaves much to be desired. Is it too much to hope that the Council will utilise the splendid material it has gathered together in its whole-time staff, and the admittedly excellent Public Health Department which it possesses, in dealing with this question of treatment? We think it will be agreed that a school doctor who confines his attention to inspection must deteriorate, however much he may keep up his knowledge by reading, unless he is given periodical opportunities to perfect himself in treatment. It is in the nature of his duties to report on treatment and its results; he is held to be a competent judge of the adequacy or otherwise of whatever treatment is given. Yet under present arrangements he is not given a chance of that practice which alone can make him perfect, and which will tend to broaden his activities and to enlarge his experience. In our view, this is a mistake, and we feel that the Council will realise that it is not in the best interests of either the Department or the schools wholly to divorce inspection from treatment. That being the case, we hope that the question of the provision of school clinics will again be brought forward in the Council, and that a more favourable decision will be given upon it.

